# Electrophysiological Correlation of the Degree of Self-Reference Effect

**DOI:** 10.1371/journal.pone.0080289

**Published:** 2013-12-02

**Authors:** Wei Fan, Jie Chen, Xiao-Yan Wang, Ronghua Cai, Qianbao Tan, Yun Chen, Qingsong Yang, Shanming Zhang, Yun Wu, Zilu Yang, Xi-Ai Wang, Yiping Zhong

**Affiliations:** 1 Department of Psychology, Hunan Normal University, Changsha, China; 2 Institute of Psychology, Hunan Normal University, Changsha, China; 3 Research Center for Psychological Development and Education, Liaoning Normal University, Liaoning, China; 4 School of Psychology and Cognitive Science, East China Normal University, Shanghai, China; University of Rome, Italy

## Abstract

The present study investigated neural correlations underlying the psychological processing of stimuli with various degrees of self-relevance. Event-related potentials were recorded for names that differ in their extent of relevance to the study participant. Participants performed a three-stimulus oddball task. ERP results showed larger P2 averaged amplitudes for highly self-relevant names than for moderately self-relevant, minimally self-relevant, and non-self-relevant names. N2 averaged amplitudes were larger for the highly self-relevant names than for the moderately self-relevant, minimally self-relevant, and non-self-relevant names. Highly self-relevant names elicited larger P3 averaged amplitudes than the moderately self-relevant names which, in turn, had larger P3 values than for minimally self-relevant names. Minimally self-relevant stimuli elicited larger P3 averaged amplitudes than non-self-relevant stimuli. These results demonstrate a degree effect of self-reference, which was indexed using electrophysiological activity.

## Introduction

The self-reference effect is the enhanced speed and quality of processing and memorization observed with respect to information that is related to the individual's self-concept [1]. Previous studies of this phenomenon showed that information is remembered better when processed in a self-referential manner than otherwise [2], [3]. A growing number of ERP studies have also found evidence of the self-relevant effect. Berlad and Pratt observed that larger P3 values are elicited by people's own names than by other words [4]. Miyakoshi and his colleagues observed that larger P3 values are elicited by participant's own objects compared with others' objects. In addition, larger P3 amplitudes are observed in response to the subject's own face than in response to either familiar or unfamiliar faces [5], [6]. Self-relevant effects indexed by enhanced P3 amplitudes also have been found in autobiographical self-relevant stimuli (e.g. the subject's phone number, the name of the subject's pet, and the name of the subject's hometown), pictures of the subject's hand, and images of the subject's handwriting [2], [7], [8].

However, previous studies have focused more on categorical differences, treating self-relevant effects as differences in behavioral or neural activation between the responses to self-relevant and non-self-relevant stimuli. They have failed to take into account the degree of self-relevance. In real-life situations, we will encounter various self-relevant information, and these self-relevant stimuli may have different level of self-relevance, and have different biological significance to individuals. Self-relevant stimuli and emotional stimuli share some similarities [8], [9]. For example, it has been found that self-relevant processing and emotional processing engage overlapping neural substrates such as nucleus acumbens and insula [9]. Moreover, self-relevant stimuli can also elicit neural activity in reward-related brain areas, e.g., ventromedial prefrontal cortex (VMPFC), ventral striatum (VS), and ventral tegmental area (VTA) [10], [11]. Yuan et al. [12], [13] found that highly emotional stimuli can elicit more brain activation than moderately emotional stimuli, and both sets of emotional stimuli elicit more brain activation than neutral stimuli. Their findings demonstrate that humans are sensitive to valence differences in negative stimuli as extremely negative stimuli represent a greater threat to survival as compared to moderately negative stimuli. Based on these considerations, it is reasonable to infer that, similar to the valence strength effect in emotional processing, the human brain would also have differential sensitivity to self-relevant stimuli of varying degree.

However, whether the brain processes highly and minimally self-relevant stimuli differently and the spatiotemporal features of the degree effect remain undetermined and merit clarification. Some studies that focused on facial recognition are relevant in this regard. Keyes, Brady, Reilly, and Foxe compared responses to the participant's own face, a friend's face and a stranger's face articulating different speech sounds [14]. They found self-face processing to differ from other processing and then discussed the differences observed with reference to EEG studies of facial processing. Scott, Luciana, Wewerka, and Nelson examined electrophysiological correlates of facial self-recognition in adults and four-year-old children [15]. Their results indicated differences in processing between adults and four-year-olds with respect to both familiar and unfamiliar faces. Four-year olds exhibited a more diffuse pattern of electrical activity than adults. Sui et al. [16] and Caharel et al. [17] also provide evidence that self-face recognition is automatic in the brains, that it occurs after face structure encoding, and that it is independent of task relevance.

Studies of the name processing are relevant here. Tacikowski et al. investigated the effects of repetition on the processing of names and faces varying in pre-experimental familiarity [18]. Results showed that participants learned faces more readily than names, possibly because faces carry more semantic information. Tacikowski et al. investigated the patterns of brain activation during the recognition of aurally and visually presented full names of the subject, a significant other, a famous person and unknown individuals [19]. This pattern of results supports the role of medial pre-frontal cortex MPFC in the processing of personally relevant information irrespective of their modality. Tateuchi et al. who used names in an orienting paradigm and found that early preattentive processing of speech sounds could be used to distinguish the subject's own name from other names irrespective of short-term stimulus context [20]. This was found to culminate in an orienting response only when subject's own name is evaluated as being contextually meaningful. Höller et al. also used self-names and found lower activity in the alpha–beta range to the participant's own name compared to other conditions. However, Höller et al. discussed their findings in terms of familiarity rather than degree of self-relevance [21].

Generally, these studies have investigated the processing of various self-related stimuli. However, some of these studies did not consider the problem of stimulus familiarity or compare materials with different degrees of familiarity, so their results cannot exclude familiarity effects. Other studies of self-related stimuli are too general and simple, and dividing self-related stimuli only into high and low groups or into related and unrelated groups. Their results cannot be used to clearly examine the degree of self-referential processing effects. Recently, Chen et al. directly investigated the self-relevant degree effect and found that more highly self-relevant stimuli elicited greater P3 amplitudes [22]. Chen et al. showed that one's own name is the most specific descriptor of the self, one's province is more general, but still more specific than China or America (which are equal in level of generality). Given that Chen et al. found that bigger P3 amplitudes were elicited by the participants' own names, followed by their province, and that there were no differences in P3 amplitudes between China and America country names, it is possible that P3 is modulated by the degree of specificity rather than the degree of self-relevance.

The present study used equally specific stimuli varying in self-relevance: the participant's own name served as the most self-relevant stimulus, his or her father's name served as the moderately self-relevant stimulus, the name of the Chairman of China as the less self-relevant stimulus, and the name of the President of the United States as the non-self-relevant stimulus.

The present study used an oddball paradigm to investigate implicit self-relevant processing by measuring high temporal resolution ERPs. Previous studies have demonstrated that the subjects' own names and faces elicit larger P3 amplitudes than did non-self-relevant names and faces [4], [5], [15], [18], [23]. Here, the P3 component was considered a valid index for self-relevant processing. For this reason, we hypothesized that P3 waves would also vary as a function of the degree of self-relevance, with higher degrees of self-relevance contributing to lager P3 amplitudes.

## Method

The experimental procedure was approved by the IRB of the Institute of Psychology, Hunan Normal University. All participants provided written informed consent before taking part in the experiment.

### 2.1 Participants

Twenty paid volunteers, all undergraduate or postgraduate students, (9 women, 11 men) aged 19–24 years (mean age: 22.5 yeas) participated in the experiment. All subjects were healthy, right-handed, had normal or corrected vision, and reported no history of cerebral injury.

### 2.2 Materials

A Chinese name typically consists of a family name and a first name and has three characters in total. For this reason, a therefore used three-character non-name lexical phrases served as the standard stimulus (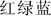), and a three-character non-lexical phrase (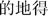) served as target stimuli. Six categories of stimuli were used in a three-stimulus oddball paradigm. Three sets of self-relevant stimuli, the non-self-relevant stimulus, and two filler stimuli, served as distracters. The name of the participant (e.g. 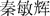) was used as the highly self-relevant stimulus, the name of participant's father (e.g. 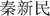) as the moderately self-relevant stimulus, the name of China's leader (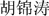) as the minimally self-relevant stimulus, and the name of the president of the United States (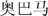), served as the non-self-relevant stimulus. All names are three Chinese characters long. Familiarity was equivalent (see below) across all sets of stimulus names. All name stimuli were made into images on a PC using Microsoft Office Picture Manager. Image size, word length, and complexity were matched across the name conditions.

### 2.3. Task design and procedures

An experimental session included a total of 800 trials in four blocks of 200 trials each. The standard stimulus was presented 560 times (70%), and the target stimulus was presented 48 times (6%). The non-self-relevant stimulus was presented 48 times (6%), and each of the three levels of self-relevant stimuli was presented 48 times (6%). The entire experiment was divided into four blocks, and the onset sequence of the stimuli was randomized across conditions in each block.

**Figure 1:**
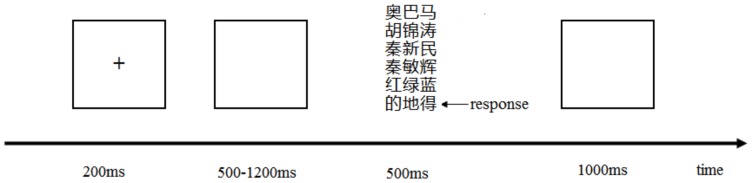
The sequence of events in an experimental trial.

Participants were seated in a quiet room approximately 100 cm from a computer screen with the horizontal and vertical visual angles all less than 5°. For the main experiment, each trial was initiated by a 200 ms presentation of a small white cross on the black computer screen. Then, a blank screen whose duration varied randomly from 500 ms to 1200 ms was followed by 500 ms presentation of a phrase from one of the six stimulus categories. After stimulus presentation, a blank screen was presented for 1000 ms. The participants' task was to observe the stimuli carefully and make a button-push response to the target stimulus (Fig. 1). To conceal the real purpose of the experiment, participants were told it was a test of reaction time. After the experiment, the participants were told their reaction time, but it was not analyzed. Between blocks, participants rested for several minutes.

Following the main experiment, in order to assess the familiarity of the stimulus and perceive self-relevance of each stimulus, participants rated stimuli as highly self-relevant, moderately self-relevant, minimally self-relevant, and non-self-relevant using a 9-point self-report covering both self relevance (1 =  “not self-related at all” to 9 =  “extremely self-related”) and familiarity (1 =  “not familiar at all” to 9 =  “extremely familiar”). The order of two rating tasks was counterbalanced across participants. Our aim was to document that self-relevance of the stimulus materials used differed ordinally and monotonically (high self-relevance > moderate self-relevance > minimal self-relevance > not self-relevant), while familiarity was statistically equivalent across those stimulus classes.

### 2.4 ERP recordings

Electroencephalograms (EEGs) were continuously recorded using 64 scalp silver/silver-chloride electrodes placed according to the international 10–20 system. All electrodes were referenced to an electrode at the left mastoid and re-referenced off-line to another electrode at the bilateral mastoid. The horizontal electrooculogram (EOG) was recorded in a bipolar manner from two electrodes placed 1.5 cm lateral to the left and right outer canthi, and the vertical EOG was recorded from electrodes below and above the left eye. The impedance for each electrode was kept below 5 kΩ. EEG was amplified (half-amplitude band pass 0.05–70 Hz) and digitized at a sampling rate of 500 Hz.

### 2.5 Data record and analysis

ERPs recorded under each set of stimulus conditions were averaged separately off-line with epochs beginning an average of 200 ms prior to and ending 600 ms after the onset of the stimulus. Trials affected by eye blinks (VEOG exceeding ±50 *μV* relative to baseline) or other artifacts (a voltage exceeding ±50 *μV* at any electrode location relative to baseline) were considered contaminated and excluded. In order to assess lateralization, the following 15 electrode sites were selected for statistical analysis: F3, FC3, C3, CP3, and P3 on the left; Fz, FCz, Cz, CPz, and Pz along the midline; and F4, FC4, C4, CP4, and P4 on the right. The average amplitudes of N1 (70–170 ms), P2 (170–270 ms), N2 (270–370 ms) and P3 (400–500 ms) were measured and analyzed at their corresponding time intervals.

Separate three-way repeated measures analyses of variance (ANOVAs) were conducted for the amplitude and latency of each component. ANOVA factors were stimulus type (4 levels: highly self-relevant, moderately self-relevant, minimally self-relevant, and non-self-relevant), laterality (3 levels: left, midline, and right) and caudality (5 levels: front, front-central, central, central-parietal, and parietal sites). The degrees of freedom of the F-ratio were corrected according to the Greenhouse–Geisser method.

## Results

### 3.1. Stimulus self-relevance and familiarity ratings

The post-experiment assessment showed a significant main effect of stimulus type in self-relevance [*F*(3, 57)  = 38.05, *P*<0.001]. Post hoc testing revealed the self-relevance scores of highly self-relevant names to be significantly higher than those of moderately self-relevant names [*F*(1, 19)  = 3.19, *P*<0.05]. These were in turn rated more self-relevant than minimally self-relevant [*F*(1, 19)  = 3.57, *P*<0.05] and non-self-relevant [*F*(1, 19)  = 8.72, *P*<0.01] names. In addition, the self-relevance scores of minimally self-relevant names were higher than those of non-self-relevant [*F*(1, 19)  = 7.37, *P*<0.01] names. In contrast, analysis of the familiarity ratings showed no significant differences across the four sets of stimuli [*F*(3, 57)  = 1.03, *P*>0.05]. The results are shown in Table 1.

**Table 1:** Four sets of experimental conditions (*M*±*SE*).

Type of assessment relevance	High relevance	Moderate relevance	Minimal relevance	No
Familiarity	8.26±1.09	8.12±1.13	8.04±1.97	7.96±1.57
Degree of self-relevance	8.32±1.93	6.52±1.87	3.54±2.32	1.38±1.06

### 3.2 ERP analysis

**Figure 2:**
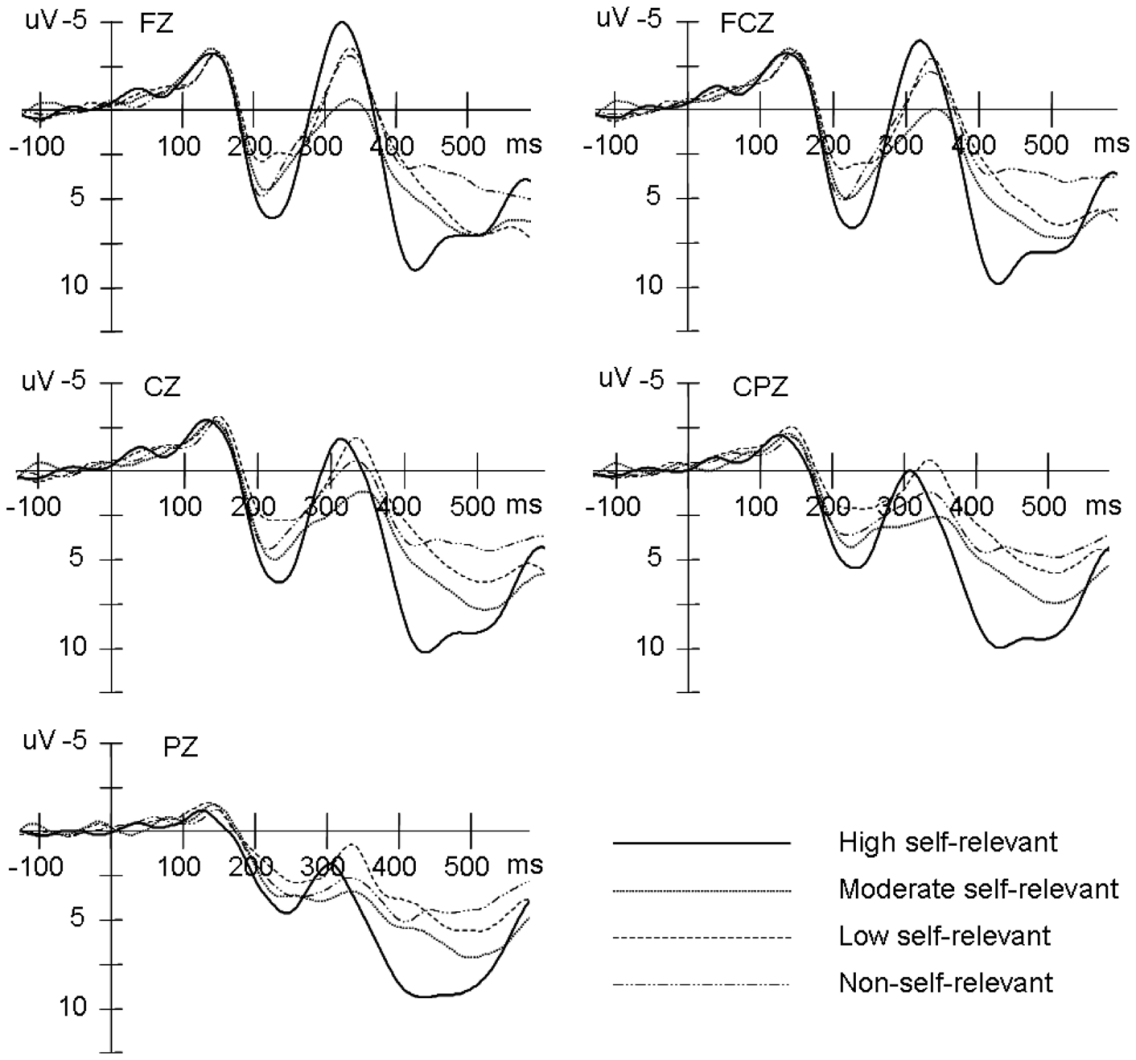
Averaged ERPs at Fz, Cz, CPz, and Pz for high self-relevant, moderate self-relevant, low self-relevant and non-self-relevant stimulus conditions.

As shown in Fig. 2, N1, P2, N2 and P3 components were elicited under each of the four sets of stimulus conditions. ANOVAs on N1(70–170 ms) averaged amplitudes [*F*(3, 57)  = 0.29, *P*>0.05] demonstrated no significant effects.

For the P2(170–270 ms) component, the ANOVA of the averaged amplitudes demonstrated a significant interaction between stimulus type and caudality [*F* (12, 228)  = 3.05, *P*<0.05]. Simple effects analysis showed that the P2 amplitudes elicited by highly self-relevant names were larger than by the other names at the front [all *F*(1, 19) >2.48, all *P*<0.05], front–central [all *F*(1,19) >3.38, all *P*<0.05] and central sites [all *F*(1,19) >3.17, all *P*<0.05]. There was also a marginally significant interaction between stimulus type and laterality [*F* (6, 114)  = 2.28, *P* = 0.07]. Simple effects analysis showed that highly self-relevant names elicited larger P2 amplitudes than the other names at the left [all *F*(1,19) >2.43, all *P*<0.05] and right sites [all *F*(1, 19) >2.52, all *P*<0.05] (Fig. 2). No other main or interaction effects were observed for the P2 component.

ANOVAs on N2 (270–370 ms) component averaged amplitudes demonstrated a significant main effect of stimulus type [*F* (3, 57)  = 2.95, *P*<0.05]. Post hoc comparison showed that highly self-relevant names evoked larger N2 amplitudes than the other names [all *F*(1, 19) >2.54, all *P*<0.05]. In addition, there was a significant interaction between stimulus type and caudality [*F* (12, 228)  = 2.88, *P*<0.05]. Simple effects analysis showed that the N2 amplitudes elicited by highly self-relevant names were larger than by the other names at the front [all *F*(1, 19) >3.88, all *P*<0.05], front–central [all *F*(1, 19) >3.63, all *P*<0.05], and central [all *F*(1,19) >3.72, all *P*<0.05] sites. A significant interaction was detected between stimulus type and laterality [*F* (6, 114)  = 4.05, *P*<0.01]. Simple effects analysis showed that the N2 amplitudes elicited by highly self-relevant names were larger than those elicited by other names at the left [all *F*(1, 19) >6.07, all *P*<0.01] and right [all *F*(1, 19) >3.13, all *P*<0.05] sites.

Regarding P3 averaged amplitude, a multiple ANOVA revealed a highly significant main effect of stimulus type [*F* (3, 57)  = 7.29, *P*<0.001]. Post hoc multiple comparison revealed that the P3 amplitudes were larger under highly self-relevant conditions than under moderately [*F*(1, 19)  = 2.79, *P*<0.05], minimally [*F*(1, 19)  = 4.51, *P*<0.001], or non-self-relevant conditions [*F*(1, 19)  = 5.27, *P*<0.001]. The moderately self-relevant names elicited larger average P3 amplitudes than the minimally self-relevant [*F*(1, 19)  = 2.37, *P*<0.05] and non-self-relevant [*F*(1, 19)  = 3.62, *P*<0.01] names, but the minimally self-relevant names elicited larger average P3 amplitudes than the non-self-relevant names [*F*(1, 19)  = 2.09, *P*<0.05] (Fig. 2). There were also significant interactions between stimulus types and laterality [*F* (6, 114)  = 6.30, *P*<0.001]. Simple effects analysis showed that P3 amplitudes elicited by highly self-relevant names were larger than those elicited by the other names at the left [all *F*(1, 19) >4.8, all *P*<0.01], middle [all *F*(1, 19) >11.46, all *P*<0.01] and right [all *F*(1, 19) >5.8, all *P*<0.01] sites.

## Discussion

In the present study, N1 activity was similar across the four types of names. This might indicate similar perceptual processing of these names. Highly self-relevant names elicited larger P2 averaged amplitudes than moderately, minimally, or non-self-relevant names. P2 activity may reflect the detection of typical stimulus features, and recruitment of attention resources [24], [25]. These results were consistent with those reported by Chen et al. and with those of several other investigations [22], [23], [26], [27]. They may indicate enhanced attention toward highly self-relevant stimuli due to their salience and biological importance. In the present study, highly self-relevant names elicited early attention and were rapidly differentiated from other names in the brain in the absence of top-bottom cognitive and controlled resources [26], [28]. This probably accounts for the larger P2 averaged amplitudes produced in response to highly self-relevant names. However, the attention effect for moderately and minimally self-relevant names was not significant with respect to the P2 component, most likely because these less self-relevant names are not as salient or motivationally important as one's own name.

In the N2 processing stage during 270–370 ms interval, the averaged N2 amplitude elicited by highly self-relevant names was found to be more negative than that elicited by other names. These results were consistent with our previous work showing that N2 amplitudes elicited by the individual's own name and national flag were more negative than those elicited by other names and flags [6], [27], [29]. Similarly, ERP studies have suggested that larger N2 amplitudes may indicate that personally familiar faces elicit stronger responses than famous faces [30], [31]. These previous studies have shown that N2 can usually be described as a non-specific component that corresponds to an attention-switching mechanism and that it is followed by a positive P300 wave. Under passive conditions, Naatanen et al. observed an N2b when the stimulus was salient enough to trigger a switch of attention [32]. In this way, the present results suggest that highly self-relevant names, due to their saliency, may automatically capture attention even if they are not targets. Nevertheless, moderately self-relevant and minimally self-relevant names elicited similar N2 amplitudes, suggesting that these self-relevant stimuli were not clearly differentiated by the brain during the N2 processing stages.

As expected, a clear P3 component was elicited by all four name conditions, and the differences in amplitude across conditions were most pronounced at the central and frontal sites. In the present study, a three-stimulus oddball task was used. Participants were required to detect the target by pressing a button in response to the target stimulus. All names served as distracters whose presentation triggered novelty processing. Accordingly, the P3 observed in this study was in fact a novelty P3 component. The novelty P3 is a known index of the late phase of orienting response, and it is sensitive to centrally controlled processes [33]–[35]. Specifically, a novelty P3 component was associated with the controlled-processing phenomena triggered by previous automatic processes [34]. Its generation has been shown to require top-down attentional mechanisms initiated by frontal lobe functions [36]. With more cognitive and controlled processing resources, the brain processed not only the self-relevance of highly and moderately self-relevant names but also the differences in the degree of self-relevance of these stimuli. Consequently, highly self-relevant names elicited larger P3 amplitudes than the moderately self-relevant names, which, in turn, elicited larger P3 amplitudes than minimally self-relevant and non-self-relevant names. In this way, in addition to the significant self-relevance effects associated with both highly self-relevant and moderately self-relevant names, the present results also demonstrated a significant self-relevance degree effect, with highly self-relevant stimuli eliciting more processing than less self-relevant stimuli. This suggested that the P3 component, unlike earlier P2 and N2 components, which reflect general processing of self information, was an effective ERP index of the self-relevance degree effect.

A subject's own name is an exclusive symbol of one's identity and is closest to the core self [37]. Numerous studies have demonstrated a processing bias for subject' own name [18], [20], [21], [27]. In the present study, subject's own name recruited greatest amount of attention and cognitive resources and may have evoked most intense emotional/motivational responses, indexed by the largest P2, N2 and P3 amplitudes induced by highly self-relevant names. In addition, the father is usually the head of the family, and the participants in the present study expressed views confirming that this held true for them. Therefore, the name of one's own father is also an important symbol of the subject's identity, and this most likely accounted for the larger P3 amplitudes associated with moderately self-relevant conditions than with minimally self-relevant and non-self-relevant conditions. Nevertheless, the self-relevance of father or state leader names was not as intense as that of people's own names. Also, one's own name, but not one's father's name, is directly indicative of him/herself. Here, highly self-relevant names elicited more cognitive processing than did moderately or minimally self-relevant names, though the self-relevant effects elicited by highly self-relevant, moderately self-relevant, and minimally self-relevant names were significant at P3 intervals.

However, self can be classified according to multiple criteria. According to the self-categorization theory, self can be classified into the individual self and the collective self. In addition, it can be also classified into the physical self and the psychological self. According to the definitions of the individual and psychological selves, all name stimuli used in the present study are more related to the individual self or the psychological self. Moreover, these name stimuli are equally specific and only varying in self-relevance. Thus, we think that the P3 effect observed in the present study should be ascribed to the extent of self-relevance instead of the other aspect of the self.

Highly self-relevant names elicited stronger P2, N2, and P3 effects than did moderately or minimally self-relevant names, with highly self-relevant names showing higher levels of activity in the midline and frontal sites. These results are consistent with the findings of previous studies. Considerable amounts of research have indicated that the cortical middle regions may play a crucial role in self-referential processing [2], [6], [27], [29]. These differences may indicate that the neural correlation underlying self-referential processing is closely related to the stimulus. For this reason, further exploration into whether this difference can be attributed to differences in the stimulus is merited.

Using ERPs technique with high-temporal resolution, the present study expanded upon previous studies by showing the degree effect of self-relevance after excluding the potential influence of specific-general continuum of experimental stimuli. Stimuli with different levels of self-relevance were found to be processed differently during both the early attentional and late cognitive processing stages. Highly self-relevant stimuli elicited attention faster than moderately self-relevant stimuli at early time points, while highly, moderately, and minimally self-relevant stimuli received different processing depths in the brain at late cognitive stages.
